# Protocol for *Fit Bodies, Fine Minds*: a randomized controlled trial on the affect of exercise and cognitive training on cognitive functioning in older adults

**DOI:** 10.1186/1471-2318-7-23

**Published:** 2007-10-04

**Authors:** Siobhan T O'Dwyer, Nicola W Burton, Nancy A Pachana, Wendy J Brown

**Affiliations:** 1 School of Human Movement Studies, University of Queensland, Australia; 2 School of Psychology, University of Queensland, Australia

## Abstract

**Background:**

Declines in cognitive functioning are a normal part of aging that can affect daily functioning and quality of life. This study will examine the impact of an exercise training program, and a combined exercise and cognitive training program, on the cognitive and physical functioning of older adults.

**Methods/Design:**

*Fit Bodies, Fine Minds *is a randomized, controlled trial. Community-dwelling adults, aged between 65 and 75 years, are randomly allocated to one of three groups for 16 weeks. The exercise-only group do three 60-minute exercise sessions per week. The exercise and cognitive training group do two 60-minute exercise sessions and one 60-minute cognitive training session per week. A no-training control group is contacted every 4 weeks. Measures of cognitive functioning, physical fitness and psychological well-being are taken at baseline (0 weeks), post-test (16 weeks) and 6-month follop (40 weeks). Qualitative responses to the program are taken at post-test.

**Discussion:**

With an increasingly aged population, interventions to improve the functioning and quality of life of older adults are particularly important. Exercise training, either alone or in combination with cognitive training, may be an effective means of optimizing cognitive functioning in older adults. This study will add to the growing evidence base on the effectiveness of these interventions.

**Trial Registration:**

Australian Clinical Trials Register: ACTRN012607000151437

## Background

Cognitive decline is a normal feature of aging. Memory difficulties, slowing mental speed, and decreased mental flexibility become evident in old age, even in older adults without dementia [1-5]. Normal, age-associated cognitive decline is related to declines in everyday functional abilities, and to increases in both formal and informal service use among older adults [6-8]. Interventions which improve cognitive functioning are therefore important for the well-being and quality of life of older adults.

### Exercise

Exercise has been suggested as an innovative approach to improving cognitive functioning in older adults. The benefits of exercise for general health and well-being in older adults are already well known [9].

Cross-sectional evidence suggests that older adults who are physically active display better cognitive functioning than their sedentary peers, in areas such as memory, reaction time, and visuospatial skills [10-13]. Prospective and longitudinal findings suggest that physical inactivity is predictive of subsequent cognitive decline and that changes in physical activity patterns over time are associated with changes in cognitive functioning [14-18].

Experimental studies of exercise training programs for older adults have generally shown improvements in cognitive functioning, particularly on measures of information processing speed and executive functioning (e.g. [19-21]). While some experimental studies have been less conclusive (e.g. [22-24]), this discrepancy is most likely attributable to methodological differences. These include differences in duration, intensity, and frequency of exercise training, along with differences in control groups, exclusion criteria, and cognitive outcome measures. A meta-analysis by Colcombe and Kramer [25] found that, overall, exercise interventions do have the potential to improve cognitive functioning in older adults, particularly mental speed and executive functioning. The meta-analysis also highlighted that the most beneficial programs are those which have exercise sessions of greater than 30 minutes duration, include aerobic *and *strength training components, and target adults aged between 65 and 70 years [25]. As this is a relatively new area of research, additional evidence is required to further support the claim that exercise training can improve cognition.

### Linking exercise and cognitive functioning

Several mechanisms have been suggested to explain the relationship between exercise and cognitive functioning. The main hypothesis is that exercise directly affects the structure and function of the brain. Increases in aerobic capacity (as a result of increases in exercise) are thought to increase cerebral blood flow, improve the transport and utilization of oxygen and glucose in the brain, promote neurogenesis (the creation of new nerve cells), and regulate neurotransmitter synthesis [14,15,17,18,26-28]. Some studies have found an association between improvements in aerobic capacity and improvements in cognitive function [19,21]. Other studies, however, have found no relationship [22-24,29]. More evidence is required to validate the idea of increased aerobic capacity as a prerequisite for improved cognitive functioning.

Psychological factors may play a role in mediating the relationship between exercise and cognition [30]. Exercise is known to improve psychological well-being [31], and psychological well-being has been associated with cognitive functioning [32,33]. A few studies of exercise training and cognitive training have included psychological measures (e.g. [22,34-38]), but results have been mixed. More research is required to elucidate the mediating role of psychological factors in the exercise-cognition relationship [30].

### Combination training

Some researchers have suggested that the benefits of exercise may be further enhanced by combining exercise training with cognitive training [36].

Cognitive training has been the traditional approach to improving cognitive functioning in older adults. It consists of learning and practicing skills and techniques to manage cognitively demanding situations (e.g. using mnemonics to aid recall). While these programs have been successful in improving the specific cognitive function targeted by the training (i.e. memory programs improve memory performance) [39-41], they do not have the potential to provide the physical *and *cognitive benefits offered by exercise training. A combination of exercise training and cognitive training, however, may provide the best of both worlds.

Fabre and colleagues [36] conducted an experimental study of combination training with four groups: aerobic training, cognitive training, aerobic *and *cognitive training, and control. They found that all three training groups improved significantly, with the combination training group (aerobic *and *cognitive training) improving significantly more than the other groups. These findings are limited, however, by the fact that the combination training group appear to have received a greater total number of training sessions per week than the other groups. Given that engaged lifestyles have been linked to cognitive performance in older adults [42-45], it is possible that the results of the combination training group could be a consequence of increased social interaction, rather than an added benefit of cognitive training. The potential of combined exercise and cognitive training could be better investigated by matching the overall training exposure of participants in a dual group to that of participants in an exercise training group.

### Feasibility

Little is known about older adults' perceptions of exercise training programs and combined exercise and cognitive training programs. While adherence and compliance data can provide some insight into the feasibility of a training program, qualitative feedback from participants provides information about satisfaction and acceptability. Collection of this type of information also provides an opportunity to identify specific strengths and weaknesses of a program, and to assess the real-life benefits of the training which cannot be measured with clinical tests or surveys [46,47].

### Aim

The aims of the present study are to:

(1) examine the impact of an exercise training program for older adults on cognitive and physical functioning,

(2) compare the impact of exercise training and a combined program of cognitive *and *exercise training for older adults (which has an equal number of total training sessions per week) on cognitive and physical functioning,

(3) examine associations between changes in aerobic capacity (as a result of exercise) and changes in cognitive functioning;

(4) examine associations between changes in psychological wellbeing (as a result of exercise) and changes in cognitive functioning; and

(5) identify older adults' perceptions of exercise training and combined exercise and cognitive training.

An exercise training program and a cognitive training program have been developed by the first author (SO), and will be evaluated in a randomized controlled trial. The content of these programs is based on previously successful training programs, and current theories regarding the relationships between exercise training, cognitive training, and cognitive functioning.

## Methods/Design

### Study design

**Figure 1:**
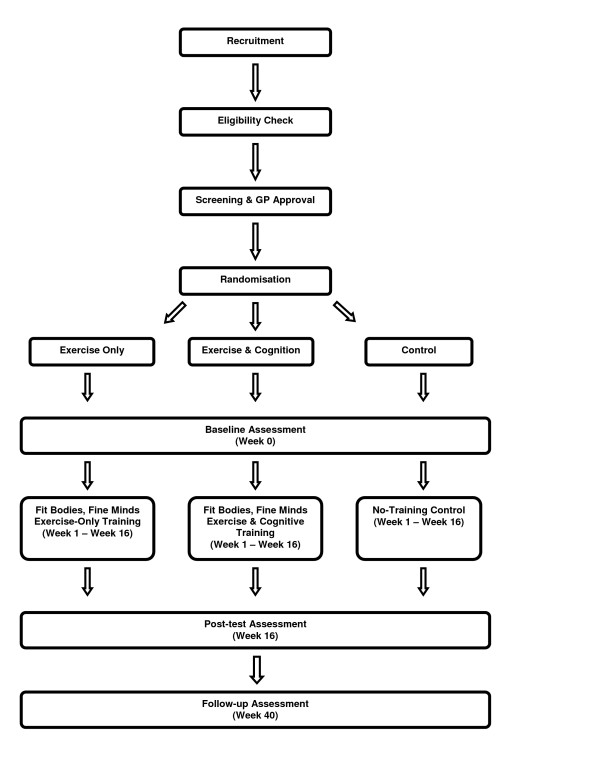
The design of the *Fit Bodies, Fine Minds *study.

*Fit Bodies, Fine Minds *is a 16-week, randomized controlled trial of exercise training and combined exercise *and *cognitive training for older adults. Participants are allocated to one of three groups: exercise-only training; exercise and cognitive training; no-training control. Assessments are conducted at baseline, post-test (16-weeks) and 6-month follow-up (40 weeks). The design is presented in Figure 1. The protocol was approved by the University of Queensland Medical Research Ethics Committee (Approval Number: HMS06/2303)

### Setting

The program is conducted at *Riverside Fitness*, a privately-owned gym in Brisbane, Australia.

### Study population

The program is designed for community-dwelling older adults without cognitive impairment. Participants are recruited through healthy aging seminars at local churches, public radio broadcasts, and the *50+ Registry *(a registry of research volunteers aged over 50 years, coordinated by the Australasian Centre on Ageing at the University of Queensland). All interested people are provided with a Participant Information Sheet detailing the nature of the program.

Individuals are initially considered eligible if they are

(1) aged between 65 and 75 years,

(2) doing less than 60 minutes of moderate-intensity exercise per week,

(3) living within 10 km of *Riverside Fitness *and the University of Queensland; and

(4) available to participate in the program during business hours.

#### Screening

Individuals who meet the initial eligibility criteria take part in a telephone interview to screen for cognitive and health problems. People who score below 21 on the Telephone Interview of Cognitive Status (TICS) [48] are excluded from participating in the program. Individuals who report more than one serious health complaint (e.g. recent heart attack, uncontrolled diabetes, or uncontrolled hypertension) are required to obtain written permission from their general practitioner (GP) to take part in the program. Those unable to obtain GP permission are excluded from participating in the program. Individuals who report diagnoses of Alzheimer's Disease, dementia, or recent head injuries are also excluded from the program.

#### Informed consent

Participants provide signed informed consent prior to baseline testing.

### Sample size

Ninety-nine participants will be recruited for the study. A sample size of 12 participants in each of the three groups would enable detection of a within-group difference, from baseline to post-test, of 1 item (SD = 1.1) on Digit Span and 184 milliseconds (SD = 198) on Choice Reaction Time. Twenty participants in each group would enable detection of a between-group difference, at post-test, of 2 items (SD = 2.2) on Digit Span. Twenty-nine participants in each group would enable detection of a between-group difference, at post-test, of 118.8 milliseconds (SD = 160) on Choice Reaction Time. These sample size calculations assume a two-tailed alpha level of 0.05 and a power of 80%. The effect sizes and standard deviations are based on results from previous studies of physical activity for cognitive functioning in older adults (e.g. [11,49]). To account for attrition over time, the required sample size (29) is increased by 10% to 33 participants per group.

### Randomization

Participants are randomly allocated using block randomization [50]. Each block contains one exercise-only group allocation, one combined exercise *and *cognitive training group allocation, and one control group allocation. The order of the groups within each block is randomly generated by selecting papers from a box. The box contains three papers marked *Exercise Only*, *Exercise & Cognition*, and *Control*, respectively. This process is repeated to generate a list of 25 blocks. Each time a participant meets the eligibility criteria and screening requirements, they are allocated to the next available place on the list.

### Blinding

For logistical reasons, participants, researchers, and instructors are not blind to the allocation of participants.

### Intervention

#### Exercise-only training

Participants in this group receive three gym-based sessions per week for 16 weeks. Each session lasts for 60 minutes and includes progressive aerobic and strength training. Each session includes a maximum of 10 participants and is supervised by at least one qualified fitness instructor.

The progression of the aerobic training is adapted from the ACSM Guidelines for Exercise Testing and Prescription [51]. Intensity is prescribed using percentage of Heart Rate Reserve (HRR). Participants on medications which attenuate heart rate (e.g. beta-blockers) are prescribed exercise intensity using Rate of Perceived Exertion (RPE; [52]). The relative progressions of intensity and duration can be seen in Table 1. Intensity is monitored with Polar A3 heat rate monitors. Participants complete the aerobic training on treadmills, stationary bicycles, cross-trainers, and stationary rowing machines.

**Table 1:** Duration and intensity progression of aerobic training

**Week**	**Duration (min)**	**Intensity (% HRR)**	**Intensity (RPE)**
1	20	50 – 60	9 – 11
2	20	50 – 60	9 – 11
3 – 5	25	60 – 70	11
6 – 8	30	60 – 70	11
9 – 11	30	70 – 80	11 – 13
12 – 14	35	70 – 80	11 – 13
15 & 16	40	75 – 85	13 – 15

Strength is assessed, and intensity prescribed, for each participant in the first gym session. Progress is monitored and prescribed by the fitness instructor. The strength training includes exercises to target major muscle groups in the upper body, lower body, and core. The upper and lower body exercises use free weights and machines. The core exercises use a swiss-ball.

Participants stretch major muscle groups at the end of each session.

#### Exercise and cognitive training

Participants in this group receive two gym-based exercise sessions and one cognitive training session per week for 16 weeks. Each session lasts for 60 minutes. The exercise training (including the progression of duration and intensity) is identical to that received in the Exercise-Only Group. The cognitive training program includes three modules, covering memory, executive functioning, and mental speed. Each session follows the same format:

(1) theoretical overview

(2) outline of 'everyday' applications

(3) practice activities

Introductory and revision sessions are conducted at the beginning and end of the program. Table 2 outlines the content of each session. A graduate psychology student leads these sessions in a quiet room away from the main gym area.

**Table 2:** Cognitive training program outline

Module	Session	Content
Introduction	1	Aging, Cognitive Functioning, Program Overview
		
Memory	2	Introduction to Memory
	3	Chunking, Organisation, Visualisation
	4	Storytelling, Method of Loci, Mnemonics
	5	Keywords, Association
		
Executive Function	6	Introduction to Reasoning
	7	Pattern Identification
	8	Concept Formation, Verbal Fluency
	9	Attention, Mental Flexibility
		
Information Processing	10	Introduction to Speed of Information Processing
	11	Simple Speed
	12	Choice Response Speed
		
Revision	13	Memory
	14	Executive Function
	15	Speed of Information Processing
	16	General

#### Control group

Participants in this group receive no formal intervention. They are contacted by telephone at Week 4 and 12 to check continued completion of the weekly diary. At Week 8 each participant is posted a cinema voucher and a letter to thank them for their ongoing involvement.

### Adherence and compliance

Attendance at each cognitive training session is recorded by the instructor. At each exercise training session, participants are provided with individual diaries in which to record the date and the intensity and duration of each activity undertaken in that session. All participants (including control participants) are provided with a weekly diary in which to record physical, social and cognitive activities undertaken outside the program.

### Assessment protocol

#### Assessment stages

In addition to screening, assessments are conducted at baseline (0 weeks), 16-weeks (post-test), and 40 weeks (6-month follow-up).

#### Modes of assessment

Screening is conducted by telephone. Cognitive and physical assessments are conducted face-to-face at the University of Queensland. Cognitive assessments are conducted by SO. Each cognitive assessment session lasts for 90 minutes. Physical assessments are conducted by an exercise physiologist and last for 30 minutes. Physical assessments are conducted immediately after the cognitive assessments. Self-administered questionnaires are used to assess psychological wellbeing. These are provided after the cognitive assessment, with participants instructed to complete them at home and mail them back within seven days using a provided reply-paid envelope. Participant perceptions of the two training programs are collected using a feedback form included with the other self-report instruments. Only training participants complete this feedback form.

### Measurements

The primary outcome measures are indicators of cognitive functioning, aerobic capacity and strength. Secondary outcome measures are anthropometric measurements, resting heart-rate, blood pressure, indicators of psychological well-being and participant perceptions. All measures are assessed at baseline, post-test, and 6-month follow-up, except for the qualitative feedback which is assessed only at post-test.

#### Screening

##### Demographics

Includes gender, age, education, occupation, nationality, marital status, and language.

##### Health information

Includes height, weight, current level of physical activity, hearing, vision, chronic diseases, illnesses, and injuries.

##### Telephone Interview of Cognitive Status (TICS-m)

This is a brief telephone-administered dementia screening measure, modified slightly from the original to be more suitable for telephone use [48,53]. Participants complete a series of short cognitive tasks, such as counting backwards, remembering a list of 10 words, and answering general knowledge questions. Performance on the original TICS has been shown to be highly correlated with performance on the Mini-Mental State Examination [48,54]. The TICS-m is sensitive and specific [53]. A score of less than 30 (out of a possible 50) on the TICS-m is the cut-off for cognitive impairment [53].

#### Primary outcome measures: Cognitive functioning

Three domains of cognitive functioning are assessed: memory, executive functioning, and speed.

##### California Verbal Learning Test (CVLT-II)

This assesses the ability to use semantic categories to aid recall (memory) [56]. Participants are presented with words from four semantic categories (e.g. fruit, tools, clothing, herbs), listed in random order. Participants must then recall these words in a number of conditions, including immediate free recall (in which participants are asked to recall as many words as they can, in any order), delayed free recall (approximately 20 minutes after the original presentation), and cued recall (in which participants are asked to recall as many words as they can which fit in each of the categories) [57]. CLVT has satisfactory test-retest reliability [57]. Norms are also available for the CVLT, which has been shown to be somewhat sensitive to the effects of aging and neuropsychological impairment [57]. Scores include the number of words correctly recalled, the number of intrusions, and the number of repetitions. To limit test-retest effects, the original form is used at baseline and 6-month follow-up, with the alternate form used at the post-test assessment (16 weeks).

##### Logical Memory

This task is from the Wechsler Memory Scale (WMS-III) [58]. It is a measure of semantic memory. Participants are presented with two brief stories and must recall information from these stories immediately and after a delay of between 10 and 20 minutes. Logical Memory has good test-retest reliability, is not susceptible to practice effects, and norms are available [57]. Logical Memory has been shown to be sensitive to the effects of aging and neuropsychological impairment [57]. Scores include the number of themes recalled and the amount of specific content information recalled [57].

##### Digit Span

This is a task from the Wechsler Memory Scale (WMS-III) [58]. It is a measure of working memory. Participants are presented with a number sequence and must immediately recall the numbers. The task progresses in difficulty as number sequences increase in length (minimum = 2 digits, maximum = 9 digits). Digit Span has two components. In the first part participants must recall the number sequences in the order presented (Digits Forward). In the second part participants must recall the number sequences in the reverse order to which they were presented (Digits Backward). Digit Span has good test-retest reliability, is not susceptible to practice effects and norms are available. Scores include the number of correctly recalled digits forward, the number of correctly recalled digits backward, and the total number of correctly recalled digits.

##### California Older Adult Stroop Test (COAST)

Stroop Tests are a common measure of executive functioning. COAST has been specially developed for use with geriatric populations [59]. It has three components. For the first task participants must identify the names of colours. For the second task participants must read colour name words. In the final task participants must identify the colour in which each colour name word is printed (e.g. the word RED is printed in green ink, so the correct participant response is "Green"). Stroop Tests generally have shown good test-retest reliability and only modest practice effects [57]. Stroop Tests have also been shown to be sensitive to the effects of aging and neuropsychological impairment, and some norms are available [57]. Scores include completion time, number of errors and number of self-corrected errors.

##### Sorting Test

This task is from the Delis-Kaplan Executive Function Scale (DKEF-S) [60]. Participants are presented with a set of six cards. The cards have words printed on them, and vary in colour and size. There are up to eight ways (rules), according to which the cards may be sorted into groups of three. In the Free Sorting condition, participants must sort the cards in as many different ways as possible and identify the rule by which they have sorted. The Sorting Test has been shown to be sensitive to the effects of aging and neuropsychological impairment [57]. The Sorting Test has good test-retest reliability and norms are available [60]. Scores include the number of correct sorts and the number of correct rule descriptions.

##### Matrix Reasoning

This is a task from the Wechsler Adult Intelligence Scale (WAIS) [61]. It is a measure of executive functioning. Participants are presented with a visual pattern that has one part missing. Participants must select, from multiple choice options, the picture which completes the pattern. The patterns progress in difficulty. Matrix Reasoning has good test-retest reliability and norms are available [61]. The score is the number of patterns correctly solved.

##### Symbol Digit Modalities Test (SDMT)

This is a measure of psychomotor speed [62]. It is a written task requiring participants to substitute numbers for symbols according to a key. Participants must complete as many substitutions as possible in 90 seconds. The SDMT has good test-retest reliability, is not susceptible to practice effects and is sensitive to the effects of aging and neuropsychological impairment. Norms are available [57]. The score is the number of correct substitutions.

##### Timed Instrumental Activities of Daily Living (TIADL)

This is a measure of applied information processing speed [63]. Participants complete five common tasks of daily living, according to set instructions. These tasks cover communication, money, food preparation, shopping, and medications, and incorporate actual everyday objects [63]. The score is based on the time taken to complete each task, with an adjustment for any errors.

##### Simple reaction time

Simple Reaction Time tasks are frequently used to measure psychomotor speed [64]. Computer-based, these tests require participants to press a button when a stimulus (e.g. a light or object) appears on the screen. Marked slowing of reaction time is evident with age and neurological impairment. Consistent with current practice [64], a Simple Reaction Time Task was developed specifically for this project. The Simple Reaction Time Task is run on a Toshiba Tecra A2 Laptop. For this task, 4 taps (faucets) are presented horizontally (and equidistant) across the screen. Participants are required to press the Space Bar when water starts to drip from one of the taps. A warning tone and message ("Get Ready") appear at the top of the screen before each trial. To avoid anticipatory responses, the interval between the warning and stimulus onset is variable. Participants complete 50 trials, with response time recorded in milliseconds from stimulus onset. Responses less than 100 millseconds are considered invalid. Scores are mean reaction time and standard deviation of reaction time.

##### Choice reaction time

Choice Reaction Time Tasks assess speed and concentration [64]. In these tests participants are presented with one stimulus and multiple response options. Responses are usually more delayed than in Simple Reaction Time Tasks, because participants must make a decision before responding [64]. The Choice Reaction Time Task was also developed specifically for this project. The Choice Reaction Time Task is run on a Toshiba Tecra A2 Laptop. For this task, 4 taps (faucets) are presented horizontally (and equidistant) across the screen. The keys D, F, J and K correspond to each tap in order from left to right. Participants are instructed that when water starts to drip from one of the taps, they are to press the corresponding key. A warning tone and a message ("Get Ready") at the top of the screen precede each trial. To avoid anticipatory responses, the interval between the warning and stimulus onset is variable. Participants complete 50 trials, with response time recorded in milliseconds from stimulus onset. Any errors are also recorded. Responses less than 100 millseconds are considered invalid. Scores are mean reaction time, standard deviation of reaction time, and number of errors.

#### Primary outcome measures: aerobic capacity and strength

##### Six-Minute Walk Test (SMWT)

This is adapted from the Senior Fitness Test [65]. It is a functional measure of aerobic endurance. A rectangular course, measuring 3 m × 15 m, is marked out for participants to walk around. Two chairs are placed inside the course for participants who need to rest during the test. The score is the number of metres walked in six minutes. The number of rests taken or adaptations used (e.g. walking sticks etc) is noted with the score.

##### Modified Astrand-Ryhming Cycle Ergometer Test

This is a multi-stage, sub-maximal exercise test, modified from the Astrand-Ryhming protocol [51]. It is conducted on a cycle-ergometer and is a measure of aerobic fitness. This test is similar to the Astrand-Ryhming protocol, except that work-rate is adjusted after the second minute, according to heart-rate and age (see Table 3). The initial power level is set at 0.5 kp for sedentary older women, and 1.0 kp for sedentary older men. This modified protocol is regularly used with clinical populations in research conducted at the School of Human Movement Studies, University of Queensland. The Astrand-Ryhming nomogram (including the age correction factor) is used to estimate maximal oxygen uptake (VO_2_max) [51].

**Table 3:** Power alterations according to age and heart rate after 2^nd ^minute (beats per minute)

	Heart rate 65 years old	Heart rate 70 years old	Heart rate 75 years old
Raise power level by			
1.0 – 1.5 kp	<92.5	<90	<87.5
0.5 – 1.0 kp	92.5 – 111.5	90 – 109	87.5 – 106.5
≤ 0.5	112.5 – 121.5	110 – 119	107.5 – 116.5
Lower power level by			
0.5 – 1.0 kp	>142.5	>140	>137.5
≤ 0.5 kp	132.5 – 141.5	130 – 139	127.5 – 136.5

##### 30-Second Chair Stand

This is taken directly from the Senior Fitness Test [65]. It is a functional measure of lower body strength. Participants are seated in a chair, with feet flat on the floor. The score is the number of full stands completed in 30 seconds, with arms folded across the chest. Any use of adaptations (e.g. pushing off the legs, using the arms of the chair) is noted with the score.

##### Arm Curl

This is taken directly from the Senior Fitness Test [65]. It is a functional measure of upper body strength. Participants are seated in a chair and given a small dumbbell (2 kg for women, 4 kg for men). The score is the number of bicep curls completed in 30 seconds. Any adaptations (e.g. use of a lighter weight) are noted with the score.

#### Secondary outcome measures: Anthropometrics and physiology

##### Anthropometrics

Measures of height (cm), weight (kg), hip girth (cm) and waist girth (cm) are taken using standard protocols [51].

##### Resting heart rate

Resting heart rate is measured by palpation of the radial artery while participants are seated. The number of beats recorded in 10 seconds is multiplied by 6, to give beats per minute.

##### Blood pressure

Diastolic and systolic blood pressure (mm Hg) are measured using standard protocols [51].

#### Secondary outcome measures: Psychological well-being

##### Memory Complaint Questionnaire (MAC-Q)

This is a six-item scale of self-reported memory decline [66]. Participants rate current memory ability against past performance for given situation (e.g. remembering the name of a person just introduced to you), on a 5-point scale from *much better now *to *much worse now*. The scale reliability is .57, with test-retest reliability of .67 [66]. Scores are summed, with double weighting for the final item (a comparison of general memory abilities). A higher score indicates greater self-reported decline.

##### SF-36: Mental Health

The Mental Health component score from the Medical Outcomes Study Short Form (SF-36; [67] reflects emotional wellbeing as measured on four subscales: vitality (items on energy and fatigue), social functioning (items on the impact of physical and emotional health on social activities), role-emotional (items on the impact of emotional problems on everyday activities), and mental health (items on happiness, nervousness and depression). The SF-36 is a widely used measure of wellbeing and Australian norms are available. A scoring algorithm is provided in the manual [68].

##### Goldberg Anxiety and Depression Scale (GADS)

This is an 18-item measure of symptoms of anxiety and depression, derived from psychiatric interview protocols [69]. Participants answer *Yes *or *No *to questions about their feelings in the past month, such as *have you been irritable? *and *have you felt hopeless? *The scale has been validated against clinical diagnoses of anxiety and depression, and found to have good specificity and sensitivity [69]. Higher scores reflect increased symptoms of anxiety and depression.

##### Health-Related Hardiness Scale (HRHS)

This scale assesses health-related hardiness, a "personality construct that moderates the stress-illness relationship" [70]. The construct is comprised of three dimensions: control, commitment, and challenge. The control dimension is associated with a sense of control over one's health and confidence in one's skills for managing health. The Control subscale of the HRHS is used in this study. Participants respond to items such as *I can avoid illness if I take care of my self*, on a 6-point scale from *strongly disagree *to *strongly agree*. The Control sub-scale reliability is .91, with test-retest reliability of .76 [70]. Higher scores reflect an increased sense of control over health.

#### Secondary outcome measures: Participant perceptions of the program

Qualitative responses about the training programs are gathered from participants to assess the suitability of the programs for older adults.

##### Program feedback form

This includes specific questions inviting participants to appraise the *Fit Bodies, Fine Minds *program. Written responses are invited on issues such as positive and negative outcomes of participation in the program, barriers to and facilitators of participation, enjoyment of activities and setting, and suitability of duration and frequency of sessions.

The form also includes two questions with 5-point Likert-scale response options. The first question is *How enjoyable was Fit Bodies, Fine Minds? *The response options are *extremely enjoyable*, *very enjoyable*, *quite enjoyable*, *somewhat enjoyable*, and *not at all enjoyable*. The second question is *How did you feel about attending Fit Bodies, Fine Minds? *The response options are *I looked forward to it ALL of the time*, *I looked forward to it MOST of the time*, *I looked forward to it SOME of the time*, *I could take it or leave it*, and *I DID NOT look forward to it at all*.

### Analyses

Analysis of variance techniques will be used to assess changes over time (baseline, post-test, follow-up), and differences between groups, on the measures of aerobic capacity and cognitive functioning. Mean scores and variability scores will be used in these analyses. Variability scores may provide a more sensitive indicator than mean scores, of changes in cognitive functioning in older adults [71]. Where appropriate age, education and gender will be included as covariates. The Reliable Change Index will be used to determine the percentage of participants who demonstrate a significant change in performance, relative to their individual baseline [72,73]. This approach may provide a more accurate picture of changes experienced by participants than simple group differences [72,73]. Regression techniques will be used to identify the influence of changes in aerobic capacity on cognitive functioning. Regressions techniques will also be used to identify the influence of changes in psychological wellbeing on cognitive functioning.

The qualitative data will be analysed to identify themes related to participant perceptions of the program.

## Discussion

"Nothing could be of greater importance to the imperatives imposed by global aging" than research into the benefits of exercise for cognition, according to Bortz [74]. This randomized controlled trial is designed to examine the effects of exercise training and combined exercise *and *cognitive training on the cognitive and physical functioning of community-dwelling older adults. It is hypothesized that

(1) both training groups will show significant improvements on measures of cognitive and physical functioning, relative to the control group,

(2) the combined training group (exercise *and *cognitive training) will show greater cognitive improvements than the exercise-only training group,

(3) improvements in cognitive functioning will be associated with improvements in aerobic capacity,

(4) improvements in cognitive functioning will be associated with improvements in psychological well-being; and

(5) participants will report positive experiences and outcomes from the two training programs.

If the training programs detailed in this protocol provide cognitive and physical benefits and are acceptable to older adults, they may represent a viable approach to improving cognitive functioning and quality of life in older adults. It is hoped that the findings from this study will add to a rapidly growing body of evidence which suggests that these types of interventions may be important for older adults, especially in a society that is facing the economic and social ramifications of an aging population.

## Abbreviations

GP: general practitioner; TICS: Telephone Interview of Cognitive Status; HRR: Heart rate reserve; ACSM: American College of Sports Medicine; RPE: Rate of Perceived Exertion; CLVT: California Verbal Learning Test; COAST: California Older Adults Stroop Test; D-KEFS: Delis-Kaplan Executive Function Scale; WMS: Wechsler Memory Scale; WAIS: Wechsler Adult Intelligence Scale; SDMT: Symbol Digit Modalities Test; TIADL: Timed Instrumental Activities of Daily Living; cm: centimeters; kg: kilograms; mm Hg: millimeters of mercury; bpm: beats per minute; SMWT: Six Minute Walk Test; kp: kiloponds; MAC-Q: Memory Complaint Questionnaire; SF-36: Medical Outcomes Study Short Form; GADS: Geriatric Anxiety and Depression Scale; HRHS: Health-related Hardiness Scale

## Competing interests

The author(s) declare that they have no competing interests.

## Authors' contributions

SO developed the idea for the study. SO, WB, NP and NB were involved in further developing the protocol. SO was responsible for drafting the manuscript and will implement the protocol and collect the data. All authors contributed to the final manuscript. All authors have read and approved the final manuscript.

## Pre-publication history

The pre-publication history for this paper can be accessed here:


